# Pneumatosis Cystoides Intestinalis after Cetuximab Chemotherapy for Squamous Cell Carcinoma of Parotid Gland

**DOI:** 10.1155/2015/530680

**Published:** 2015-01-31

**Authors:** Christos Petrides, Neofytou Kyriakos, Ioannou Andreas, Parpounas Konstantinos, Georgiou Chrysanthos, Petrou Athanasios, Emmanouil Pikoulis

**Affiliations:** ^1^Department of Surgery, Nicosia Government Hospital, Palaios Dromos Lefkosias-Lemesou, No. 215, Strovolos, 2029 Nicosia, Cyprus; ^2^Department of Academic Surgery, Royal Marsden Hospital, Upper GI/HPB Unit, Fulham Road, London SW3 6JJ, UK; ^3^Department of Pathology, Nicosia Government Hospital, Palaios Dromos Lefkosias-Lemesou, No. 215, Strovolos, 2029 Nicosia, Cyprus; ^4^Laiko General Hospital, Athens Medical School, National and Kapodistrian University, Agiou Thoma 17, 11527 Athens, Greece

## Abstract

Pneumatosis intestinalis, defined as gas in the bowel wall, is often first identified on abdominal radiographs or computed tomography (CT) scans. It is a radiographic finding and not a diagnosis, as the etiology varies from benign conditions to fulminant gastrointestinal disease. We report here a case of pneumatosis intestinalis associated with cetuximab therapy for squamous cell carcinoma of head and neck. The patient underwent laparotomy based on the CT scan and the result was pneumatosis intestinalis without any signs of necrotizing enterocolitis.

## 1. Introduction

Cetuximab is a chemotherapeutic agent that belongs to the group of chimeric monoclonal antibodies of epidermal growth factor receptor (EGFR) and has a significant role in clinical oncology regimens. Its action involves binding with high affinity to the extracellular domain of human EGFR and blocking ligand binding, resulting in the inhibition of the receptor function. Its action includes furthermore the targeting of EGFR-expressing tumor cells for cytotoxic immune cells [1]. With regard to head and neck cancer, the sixth most common cancer worldwide (5% of all malignancies), cetuximab has a major role since EGFR is almost invariably expressed in squamous cell cancers of head and neck (SCCHN) and this overexpression is associated with more aggressive disease and poorer prognosis. Cetuximab has been evaluated in combination with radiotherapy, chemoradiotherapy, and induction chemotherapies and was found to be superior without significantly raising the toxicity level of the combined treatment. More specifically cetuximab was found to improve overall survival, progression-free survival, and time to local recurrence when compared to radiotherapy alone in cases of locally advanced squamous cell carcinoma of head and neck (LA SCCHN) [2].

It can be used in combination with radiation therapy for the initial treatment of locally or regionally advanced squamous cell carcinoma of head and neck or in combination with platinum-based therapy with 5-FU for the first-line treatment of patients with recurrent locoregional disease or metastatic squamous cell carcinoma of head and neck. As a single agent, is indicated for the treatment of patients with recurrent or metastatic squamous cell carcinoma of head and neck for whom prior platinum-based therapy has failed [3, 4].

We report a case of pneumatosis cystoides intestinalis (PCI) in a patient who received first-line adjuvant chemotherapy with 5-FU and cisplatin followed by second line chemotherapy with Methotrexate after undergoing an extensive regional R1-resection of a SCC lesion on his right ear followed by parotidectomy and level II lymph node resection due to relapse of SCC to the right parotid gland. The patient during this period receives only cetuximab therapy and there are only three more cases of cetuximab-induced or cetuximab-related PCI (in combination with other chemotherapeutic agents) that have been reported in the literature, during chemotherapy for advanced colorectal cancer [5–7].

## 2. Case Report

An 83-year-old male underwent an extensive regional R1-resection for a SCC lesion on his right ear and reconstruction with removable skin flap in 2012, followed by parotidectomy six months later and level II lymph node resection due to relapse of the SCC to the right parotid gland. He received an adjuvant first-line chemotherapy and radiotherapy (9 cycles) with 5-FU, cisplatin and second line chemotherapy with Methotrexate 8 mg weekly, with initial good response to treatment. Unfortunately, a few months later his disease started to progress. The patient is now on chemotherapy with cetuximab (last dose 36 hours ago), without any distal metastases but with regional outspread of the disease to his right ear and mandible.

**Figure 1:**
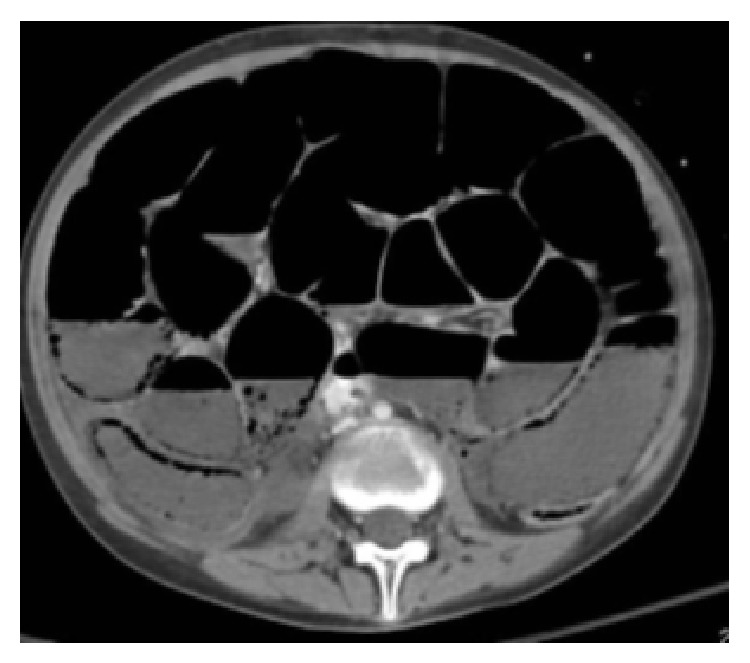
Computed tomography showing air in the intestinal wall.

The patient presented to the Emergency Department of Nicosia General Hospital with epigastric pain and vomiting during the last five days, without bloody stools. The patient was afebrile, with mild abdominal distention and without peritoneal signs. Leukocytes, amylase, and lactic acid were all within the normal ranges. Abdominal radiograph revealed free air intraperitoneally and abdominal/pelvic computed tomography revealed findings of small bowel ischemic necrosis in the presence of free intraperitoneal air and air in the intestinal wall (Figure 1).

The clinical suspicion of bowel perforation was part of the differential diagnosis and the decision of an emergency surgical operation was made. Emergency exploratory laparotomy revealed a part of small intestine (2 m) with ischemic signs, including air bubbles and bowel edema but the bowel was considered to be viable and no resection was performed. Thorough exploration of peritoneal cavity did not reveal any other pathological signs or intestinal leak. We concluded that the findings could only be associated with the pathogenesis of pneumatosis intestinalis.

Two drainage tubes were placed and the patient was sent to ICU for postsurgery follow-up and weaning. After 24 hours the patient was transferred to surgical ward for further treatment. After six days of hospitalization and complete resolution of symptoms the patient was discharged.

## 3. Discussion

**Figure 2:**
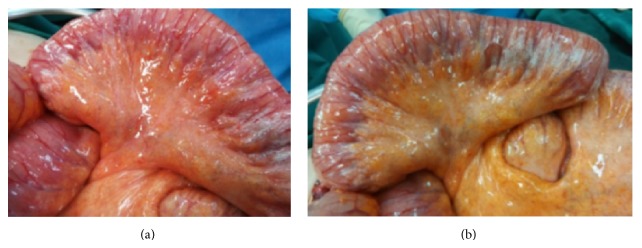
Intraoperative images of pneumatosis intestinalis.

The pathogenesis of pneumatosis intestinalis remains unclear, but the process may involve loss of mucosal integrity, increased intraluminal pressure, and finally increased intraluminal gas production as a result of bacterial overgrowth [6, 7]. Various predisposing factors have been reported associated with pneumatosis intestinalis, including trauma, inflammatory diseases, autoimmune diseases, pulmonary causes, celiac disease, leukemia, amyloidosis, and last drugs. Our patient had no known risk factors other than administration of chemotherapy with cetuximab. Pneumatosis intestinalis occurs in two forms. Primary pneumatosis intestinalis (15% of cases) is a benign idiopathic condition in which multiple thin-walled cysts develop in the submucosa or subserosa of the colon. Usually, this form has no associated symptoms, and the cysts may be found incidentally through radiography or endoscopy. When the cysts protrude into the lumen, they may mimic polyps or carcinomas, as shown on barium enema studies. This primary form is often termed pneumatosis cystoides intestinalis [8]. The secondary form is associated with obstructive pulmonary disease, as well as with obstructive and necrotic gastrointestinal disease. Microvesicular gas collections, defined as 10–100 mm cysts or bubbles within the lamina propria, are predominantly associated with primary (benign) pneumatosis intestinalis, whereas linear or curvilinear gas collections seen parallel to the bowel wall are found in secondary pneumatosis. Therefore, linear gas collections are usually an ominous sign (Figure 2) [9].

Our patient had no signs of peritonitis symptoms and pneumatosis intestinalis was found during CT scan. This finding suggests the need for careful clinical follow-up and a high level of suspicion of pneumatosis intestinalis for these patients and a less aggressive approach as concern of surgery. The possibility of bowel perforation and the resulting sepsis was the significant factor that worried us and led to the decision of operation. Surgery should only be performed in patients who are not responding to nonoperative treatment, especially those with signs of perforation, peritonitis, or abdominal sepsis. Some authors suggest that metabolic acidosis, elevated lactic acid, elevated amylase, and portal vein gas should be considered as indications for surgery.

## 4. Conclusion

We experienced a rare case of pneumatosis intestinalis following chemotherapy with cetuximab agent. Although this emergency condition leads us to surgery, a more conservative approach could be the initial treatment for these patients.
